# Association of Hyperinflammatory Subphenotype With Code Status De-Escalation in Patients With Acute Respiratory Failure

**DOI:** 10.1016/j.chstcc.2024.100098

**Published:** 2024-09-10

**Authors:** Amanda C. Moale, S. Mehdi Nouraie, Haris Zia, Caitlin Schaefer, Ian J. Barbash, Douglas B. White, Bryan J. McVerry, Georgios D. Kitsios

**Affiliations:** Division of Pulmonary, Allergy, Critical Care, and Sleep Medicine (A. C. M., S. M. N., C. S., I. J. B., B. J. M., and G. D. K.), University of Pittsburgh School of Medicine; the Department of Critical Care Medicine (I. J. B., D. B. W., and B. J. M.), University of Pittsburgh School of Medicine; and the Department of Medicine (H. Z.), University of Kentucky.

To the Editor:

Hyperinflammatory and hypoinflammatory subphenotypes in critical illness can predict clinical outcomes and may identify patient subgroups with differential treatment responses.^1–4^ Initially derived in ARDS, plasma biomarker-based subphenotypes have been replicated in mechanically ventilated patients with acute respiratory failure (ARF) resulting from different causes, even if they do not meet ARDS criteria.^2,3^

Given the divergent outcomes and treatment responses among subphenotypes, emphasis is growing on bedside identification of patients’ biological subphenotypes to inform therapeutic approaches and prognosis.^3^ However, it is unclear whether clinical judgment of illness severity and prognostication reliably align with biological subphenotypes. Code status de-escalation (CSD) often occurs in response to clinical deterioration or anticipated poor outcomes.^5^ Thus, considering CSD as a proxy for clinical judgment of illness severity, we hypothesized that the hyperinflammatory subphenotype would be associated with CSD, given that patients with the hyperinflammatory subphenotype are known to experience worse clinical outcomes. In secondary analyses, we examined associations between subphenotypes and survival in patient subgroups defined by the final code status during ICU stay.

## Methods

### Data Source

We analyzed data from a cohort of mechanically ventilated adult patients with ARF, enrolled between October 2011 and January 2019. Subtypes of ARF included ARDS per Berlin definition, at-risk for ARDS, congestive heart failure, acute-on-chronic respiratory failure, and intubation for airway protection.^2^ The inclusion and exclusion criteria, sample collections, biomarker variables, subphenotype classifications, and outcomes were detailed previously.^2,3^ We collected baseline characteristics, diagnoses, ventilatory and laboratory parameters, as well as code status data during ICU stay. Our medical ICU fosters regular family support and longitudinal communication among nurse partners, families, and the medical team.^6^ The study was approved by the University of Pittsburgh Institutional Review Board (Identifier: STUDY19050099). All participants or their surrogates provided informed consent.

### Data Analysis

We classified baseline subphenotypes, within 72 hours of intubation, with a parsimonious logistic regression model using four plasma biomarkers.^2,3^ Clinicians were unaware of subphenotype classifications. We defined CSD as a change from full-on admission to a limited code status (nonfull) during the ICU course, including do not resuscitate (DNR), do not intubate (DNI) and DNR, or comfort measures only (CMO). We focused on patients with full code on ICU admission, because limited code status on admission may influence physicians’ decisions regarding invasive care and non-CPR treatments.^7^ As our primary outcome, we retrospectively investigated the association between subphenotypes and CSD with unadjusted and adjusted logistic regression models, expressed as ORs with 95% CIs. We selected covariates for adjusted analyses based on preexisting knowledge, but did not adjust for illness severity because organ failure components were included as variables in subphenotype derivation via latent class analysis.^2,3^ In secondary time-to-event analyses, we examined the associations between (1) subphenotypes and time to CSD from ICU admission and (2) subphenotypes and 90-day survival in subgroups defined by the final code status (full, DNR or DNR and DNI, and CMO) using Kaplan-Meier survival curves and log-rank tests. We performed all analyses in STATA version 18 software (StataCorp LLC) and R software ggsankey and survival packages (R Foundation for Statistical Computing).

## Results

**Figure 1 –:**
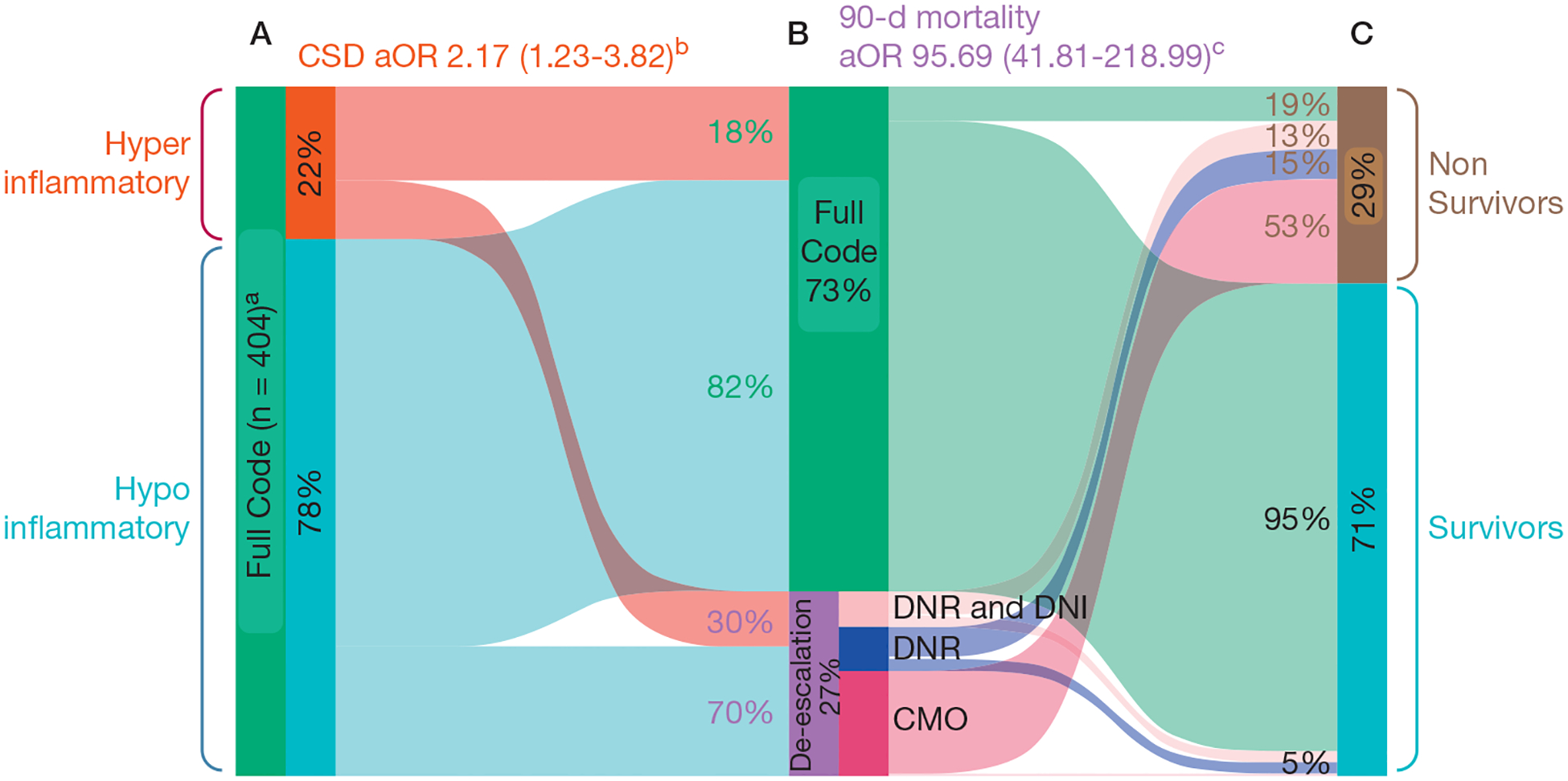
Sankey diagram showing CSD and survivorship. A, Intubated patients with acute respiratory failure and designated as full code on admission (N = 404) were classified retrospectively into hyperinflammatory (n = 84 [21.5%]; red) and hypoinflammatory (n = 306 [78.5%]; light blue) phenotypes at baseline by a previously published parsimonious model.^3^ B, CSD during the ICU course occurred in 108 patients (26.7%; purple bar) and was more frequent in the hyperinflammatory group compared with the hypoinflammatory group (crude OR, 1.90 [95% CI, 1.14–3.16]; aOR, 2.17 [95% CI, 1.23–3.82]; *P* = .008). C, At 90 days after admission, 116 patients had died (28.7%; brown bar), with percentages of the last recorded code statuses displayed. CSD during the ICU course was associated with increased mortality (crude OR, 83.62 [95% CI, 41.12–170.06]; aOR, 95.69 [95% CI, 41.81–218.99]; *P* < .001). ^a^Subphenotype data missing from 14 participants. ^b^Adjusted for age and comorbidities, including chronic cardiac failure, chronic kidney disease, COPD, diabetes, and pulmonary fibrosis. ^c^Adjusted for age, comorbidities, and subphenotype classification. aOR = adjusted OR; CMO = comfort measures only; CSD = code status de-escalation; DNI = do not intubate; DNR = do not resuscitate.

**Figure 2 –:**
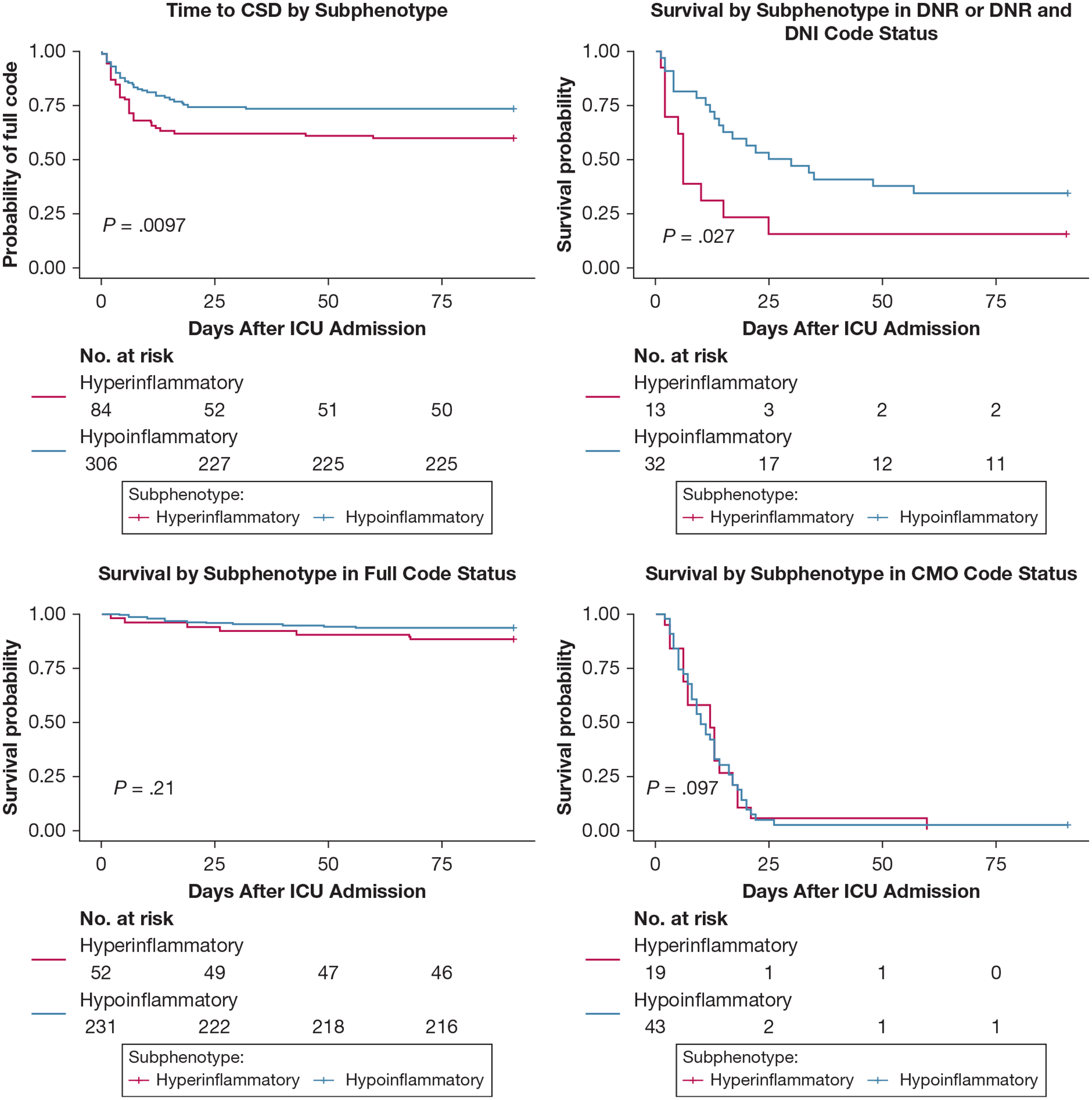
A-D, Graphs showing time-to-event analysis of baseline subphenotypes for the outcomes of CSD, and 90-day survival. Results are displayed as Kaplan-Meier curves with log-rank tests for significant differences (*P* < .05) between hyperinflammatory (red line) and hypoinflammatory subphenotypes (blue line). A, Patients in the hyperinflammatory subphenotype showed shorter time to CSD compared with patients in the hypoinflammatory subphenotype. B-D, Stratified analyses by final code status, grouped as full (B), DNR or DNR and DNI (C), and CMO (D), showed that patients in the hyperinflammatory subphenotype who experienced CSD to DNR or DNR and DNI experienced worse 90-day survival from last code status determination compared with patients in the hypoinflammatory subphenotype (C), whereas no survival differences by subphenotype were seen for patients who either remained full code or de-escalated to CMO. CSD = code status de-escalation; CMO = comfort measures only; DNI = do not intubate; DNR = do not resuscitate.

We enrolled 433 patients with ARF during the study period and analyzed data from 404 patients with full code status on admission. Code status was deescalated in 108 patients (26.7%) during the ICU course (Table 1). Patients with the hyperinflammatory subphenotype (21.5%) showed a statistically significant association with CSD compared with patients with the hypoinflammatory subphenotype in both unadjusted (OR, 1.90; 95% CI, 1.14–3.16) and adjusted (OR, 2.17; 95% CI, 1.23–3.82) analyses for age and comorbidities (Fig 1). Time to CSD also was shorter for patients with the hyperinflammatory subphenotype (*P* = .010) (Fig 2), and results were unchanged in sensitivity analyses limited to patients who experienced CSD ≥ 72 hours after ICU admission. Among patients who underwent CSD, patients with the hyperinflammatory subphenotype showed higher illness severity by Sequential Organ Failure Assessment scores (*P* < .001) and worse renal function, but otherwise similar baseline characteristics as patients with the hypoinflammatory subphenotype (Table 1). Similarly, among patients who remained full code, patients with the hyperinflammatory subphenotype showed higher Sequential Organ Failure Assessment scores, worse renal function, and more frequent shock, but an otherwise similar burden of comorbid conditions and age as patients with the hypoinflammatory subphenotype (Table 1).

Patients who experienced CSD also showed a statistically significant association with 90-day mortality in both unadjusted (OR, 83.62; 95% CI, 41.12–170.06) and adjusted (OR, 95.69; 95% CI, 41.81–218.99) analyses for age, comorbidities, and subphenotypes (Fig 1). Among nonsurvivors at 90 days, the last recorded code status was full (n = 22 [19.0%]), DNR and DNI (n = 15 [12.9%]), DNR (n = 18 [15.5%]), and CMO (n = 61) [52.6%]) (Fig 1). When stratified by final code status, the hyperinflammatory subphenotype showed worse 90-day survival among patients with CSD to either DNR or DNR and DNI (*P* = .027) (Fig 2), but no differences among those who remained full code or experienced CSD to CMO (Fig 2).

## Discussion

We identified an independent association between biological subphenotypes and CSD. Our findings revealed a statistically significant association between the hyperinflammatory subphenotype and CSD, as well as a shorter time to occurrence compared with patients with the hypoinflammatory subphenotype with ARF.

Because clinicians were unaware of patients’ subphenotype classifications at the time of clinical care, our findings suggest that clinicians likely gauged illness severity and prognosis effectively and that their impressions may correlate with the underlying biological subphenotype. However, cross-sectional biological subphenotypes cannot capture the dynamic evolution of critical illness.^8^ Thus, longitudinal subphenotyping could identify better patient subgroups with distinct illness trajectories who may respond differently to targeted interventions throughout the critical illness continuum.

Furthermore, most patients who experienced CSD died within 90 days. Although nearly all who transitioned to CMO died regardless of subphenotype, among those who de-escalated to DNR or DNR and DNI, patients with the hyperinflammatory subphenotype showed significantly worse survival compared with patients with the hypoinflammatory subphenotype. Thus, although CSD may indicate heightened illness severity and potentially may worsen survival independently, patients with the hyperinflammatory subphenotype still fare worse than patients with the hypoinflammatory subphenotype after transitioning to DNR or DNR and DNI.

Our study is limited by its retrospective single-center design and sample size. Nevertheless, the similar frequency and discriminatory variables of the two subphenotypes to those described previously support the generalizability of our results.^2^ Our study cannot draw any conclusions on causality and lacks clarity on how illness severity influenced code status conversations, because such information was not captured in the electronic medical record. Future research should seek to assess clinicians’ ability to predict subphenotypes, as well as the impact of biological subphenotype knowledge on medical decision-making as it pertains to CSD.^5^ Although bedside biological phenotyping holds promise, caution must be exercised when applying prognostic predictions for individual patients. Misclassifications or misinterpretation of data could affect counseling with patients and families inadvertently regarding critical illness outcomes and care goals.^8^

## Conclusions

CSD was statistically significant and occurred sooner in mechanically ventilated patients with ARF who were classified retrospectively to the hyperinflammatory subphenotype compared with those classified to the hypoinflammatory subphenotype, suggesting that clinicians’ overall assessment may correlate with baseline biological subphenotypes. Prospective bedside identification of biological data may have implications for prognostication and shared decision-making in the ICU that warrant further investigation and careful consideration.

## Figures and Tables

**TABLE 1]:** Participant Characteristics Categorized by Subphenotype and CSD Status From Full Code Status on Admission

Variable	Total Cohort	CSD^a^					
		No^b^			Yes^c^		
		Total	Hyperinflammatory	Hypoinflammatory	Total	Hyperinflammatory	Hypoinflammatory
No. of patients	404	296	52	231	108	32	75
Age, y	57.1 (46.1–66.3)	55.4 (44.0–64.1)	53.5 (38.8–62.9)	55.5 (45.0–64.4)	63.6 (55.4–70.6)	62.2 (56.0–70.3)	64.2 (55.3–70.7)
Sex							
Male	219 (54.2)	157 (53.0)	33 (63.5)	116 (50.2)	62 (57.4)	21 (65.6)	40 (53.3)
Female	185 (45.8)	139 (47.0)	19 (36.5)	115 (49.8)	46 (42.6)	11 (34.4)	35 (46.7)
BMI, kg/m^2^	28.8 (25.0–36.3)	29.5 (25.0–36.0)	29.4 (24.9–34.1)	29.5 (25.4–36.6)	28.4 (25.0–36.5)	28.3 (26.0–36.6)	28.4 (24.7–36.5)
Race							
White	370 (91.6)	269 (90.9)	46 (88.5)	212 (91.8)	101 (93.5)	28 (87.5)	72 (96.0)
Black	34 (8.4)	27 (9.1)	6 (11.5)	19 (8.2)	7 (6.5)	4 (12.5)	3 (4.0)
Chronic disease							
Chronic cardiac failure	48 (11.9)	32 (10.8)	4 (7.7)	26 (11.3)	16 (14.8)	2 (6.3)	14 (18.7)
Chronic kidney disease	62 (15.4)	42 (14.2)	15 (28.9)	25 (10.8)	20 (18.5)	10 (31.3)	10 (13.3)
COPD	93 (23.0)	66 (22.3)	10 (19.2)	51 (22.1)	27 (25.0)	7 (21.9)	20 (26.7)
Pulmonary fibrosis	21 (5.2)	10 (3.4)	3 (5.8)	6 (2.6)	11 (10.2)	2 (6.3)	9 (12.0)
Diabetes	129 (31.9)	96 (32.4)	17 (32.7)	75 (32.5)	33 (30.6)	12 (37.5)	20 (26.7)
Active neoplasms	19 (4.7)	7 (2.4)	2 (3.9)	5 (2.2)	12 (11.1)	3 (9.4)	9 (12.0)
Immunosuppression	85 (21.0)	58 (19.6)	10 (19.2)	44 (19.1)	27 (25.0)	10 (31.3)	16 (21.3)
Acute respiratory failure subtype							
ARDS	110 (27.2)	79 (26.7)	20 (38.5)	54 (23.4)	31 (28.7)	12 (37.5)	19 (25.3)
At risk of ARDS	157 (38.9)	122 (41.2)	24 (46.2)	93 (40.3)	35 (32.4)	14 (43.8)	21 (28.0)
Congestive heart failure	34 (8.4)	21 (7.1)	4 (7.7)	17 (7.7)	13 (12.0)	2 (6.3)	10 (13.3)
Acute-on-chronic respiratory failure	25 (6.2)	10 (3.4)	0 (0.0)	10 (4.3)	15 (13.9)	1 (3.1)	14 (18.7)
Intubation for airway protection	56 (13.9)	49 (16.5)	2 (3.9)	44 (19.1)	7 (6.5)	2 (6.3)	5 (6.7)
Other	22 (5.4)	15 (5.1)	2 (3.9)	13 (5.6)	7 (6.5)	1 (3.1)	6 (8.0)
Mechanical ventilator variables							
Worst Pao_2_ to Fio_2_ ratio	164 (116.5–210)	166.5 (117–211)	156.5 (102.5–240.5)	168 (117–212)	164 (110.5–208)	175 (126–234)	150 (102–205)
Peak inspiratory pressure, cm H_2_0	26 (20–31)	25 (19–30)	27 (17–32)	25 (19–30)	26.5 (21–32)	26.5 (21.5–32)	26 (21–33)
Tidal volume, mL/kg predicted body weight	6.6 (6.0–7.5)	6.6 (6.0–7.5)	6.7 (6.0–7.5)	6.6 (6.0–7.5)	6.4 (5.8–7.3)	6.8 (6.0–7.7)	6.3 (5.7–7.3)
Severity of illness and clinical outcomes							
Shock requiring vasopressors	175 (43.3)	111 (37.5)	34 (65.4)	72 (31.2)	64 (59.3)	24 (75.0)	40 (53.3)
AKI	263 (65.1)	183 (61.8)	46 (88.5)	133 (57.6)	80 (74.1)	30 (93.8)	49 (65.3)
SOFA score	7 (4–9)	7 (4–9)	10 (8–11)	6 (4–8)	8 (5–10)	11 (7.5–13)	7 (5–10)
Mortality							
30 d	103 (25.5)	14 (4.7)	4 (7.7)	10 (4.3)	89 (82.4)	29 (90.6)	59 (78.7)
90 d	116 (28.7)	22 (7.4)	6 (11.5)	15 (6.5)	94 (87.0)	30 (93.8)	63 (84.0)
ICU length of stay	8 (4–13)	7 (4–12)	10 (6–16.5)	7 (4–11)	9.5 (5–15)	6.5 (5–12.5)	10 (5–17)
Ventilator-free days at 28 d	21 (4–25)	23 (18–25)	22 (14–25)	23 (19–25)	0 (0–17.5)	0 (0–2.5)	0 (0–18)
Hyperinflammatory phenotype	84 (21.5)^d^	52 (18.4)^e^	NA	NA	32 (29.9)^f^	NA	NA
Palliative care consultation	23 (5.7)	6 (2.0)	0 (0.0)	5 (2.2)	17 (15.7)	5 (15.6)	12 (16.0)

Data are presented as No. (%) or median (interquartile range) unless otherwise indicated. AKI = acute kidney injury; CSD = code status de-escalation; CMO = comfort measures only; DNI = do not intubate; DNR = do not resuscitate; NA = not applicable; SOFA = Sequential Organ Failure Assessment.

a Subphenotype data missing from 14 participants.

b All remained full code status: n = 296 (100%).

c Last recorded code status: CMO, 62 (57.4%); DNR, 25 (23.2%); and DNR and DNI, 21 (19.4%).

d Data missing from 14 participants.

e Data missing from 13 participants.

f Data missing from 1 participant.
